# Anæsthetics and Narcotics

**Published:** 1881-04

**Authors:** Laurence Turnbull

**Affiliations:** Aural Surgeon to Jefferson Medical College Hospital, Philadelpia


ARTICLE II.

### ANÆSTHETICS AND NARCOTICS.

BY LAURENCE TURNBULL, M. D.,
Aural Surgeon to Jefferson Medical College Hospital, Philadelpia.

At no time that we remember has the profession evinced so much interest in this most important subject of anæsthetics, or been so desirous of being fully informed of the nature, properties, and above all, of the dangers in the use of this class of medicinal agents.

There are certain new agents that still require more light shed upon their effect, and there are some of the more dangerous that require to be kept before the mind of the profession as such, else valuable lives will be lost, and the happiness both of the immediate families and the community at large, suffer. We should look upon all anæsthetics as dangerous and poisonous agents, like opium or its salts, or like all the class of true hypnotics, and to be employed in medicine with the same care, and with all the necessary precautions.

Do the effects of chloroform and its compounds, or the various ethers employed for anæsthetic purposes by inhalation, differ materially from the class of remedies we call narcotics? No. Have we yet found a medicinal preparation that will place the nervous, vascular and muscular system in such perfect and absolute repose that the knife may cut the flesh, and the forceps crush the bones, without the system showing any evidence of pain, and this without, at the same time, causing more or less risk of death from the sudden arrest of the respiration or circulation? We say, no.

Has the medical profession in this country, either collectively or individually, contributed or offered any prize, award, testimonial, or money to the original individuals who discovered or made the first use, for the benefit of the medical profession, of anæsthetic agents, or has there been as in Great Britain, any special grant of means by which original investigation could be carried on by a committee in regard to the nature, action, or chemical composition of any of these agents? We say, no!

Is there any laboratory where a physician can find the necessary animals, appliances in this or any other city in the United States by which, at a moderate outlay of time and money, he can make original observations and experiments? We have not found such an one.

Is it just, fair or generous in men who for years have thought, studied or written on the subject of anæsthetics, and having the requisite knowledge, skill and gift of writing with ease, elegance and fluency, to do so little in the way of original work, but only to review the work of others, find fault with the very mistakes they make themselves, namely—typographical errors—and make unfair comparisons between the labor of paid men, of one of the oldest nations of the world, and one of the new; or between one who has at his command, at a very moderate cost, all the appliances necessary to undertake the most delicate and minute investigations and researches; with publishers willing and generous enough to publish such elaborate works, tables, etc., without inquiring first cui bono, will it pay? Strange as it may sound, we know to our cost that publishers absolutely refused to publish works of this class in this country, and the authors had to pay for it themselves after all their mental and physical labor. Again, these erudite reviewers praise to the skies the works of those devoted men who write in a foreign tongue (French, German or Italian) which they place at the head of their reviews, yet will not undertake the labor of translating the whole work for the benefit of the general practitioner, but cull from its pages fragments that will suit their own peculiar views and purposes.

We consider the present time pregnant with new facts, observation and experiments which should not be buried but should be brought fresh and early to the notice of the general profession before being lost or overwhelmed with a new series in this age of medical progress.

We should first state that in what we attempted in 1876–7–8–9–80, we consider we have it in part accomplished, namely—that we have proven at great labor and expense in experimenting, collecting and arranging statistics, etc., that in chloroform, pure and simple, as an anæsthetic, confidence "is most thoroughly shaken," and that it is only to be employed when no other anæsthetic agent can possibly take its place, and with all the care and caution we can use in administering this, the most powerful narcotic agent, to be found in the list of the materia medica.

At no other time has pure ether been so much employed and appreciated, not only in this country, but in England. This was in part brought about by our work in this country and by the publication of the various accidents from chloroform in the *British Medical Journal*; also a knowledge of the proper mode of its use by Dr. J. Jefferies, of Boston. It is very evident from the whole character and scope of a recent elaborate review by Dr. Reeves, of Ohio, *American Journal of Medical Science*, July, 1880, that his object was to bolster up chloroform, and at the end of the paper finding that he had no new facts to go upon in regard to its safety so he endeavors to retain the agent to use in the cases of women and children. When Dr. Reeves was desired to inform Dr Young " whether he knew of any young children, for instance, under the age of ten or twelve years, dying under the influence of chloroform, he stated, "of 110 cases to which we have alluded there were only two of children under five years of age.—*Cincinnati Lancet and Clinic*, Oct. 30, 1880. Is this the true record? In our work we have fully proven that chloroform is fatal in certain cases to children. Turn to our work on anæsthetics and you will find eighteen fatal cases of death of children from chloroform. A fatal case is also mentioned in the *Medical Gazette*, March 7th, and the *London Lancet* reports two deaths in 1867, and still another in this country: A very sad accident, resulting in the death of Elfride Mary Catharine, the only child of Prof. Frederick R. Houry, of the Yale Art School. occurred in New Haven. Professor Houry left his home to attend a Christmas service. Mrs. Houry had been ailing for some time and had been using chloroform, keeping the bottle in a closet. She fell asleep in her chair, and the child, lacking only a month of being two years old, crept to the closet and obtained the bottle of chloroform, removing the cork and inhaling the contents. When the Professor returned to his home his wife was yet asleep in her chair and the child was dead on the floor.—*New Haven*, Jan. 1881. In a case reported by Richard L. Macdonnell, B. A., M. D., *Canada Medical and Surgical Journal*, occurring January 1880, of almost fatal poisoning from chloroform, the patient received altogether the vapor of only thirty minims. In a child of only two and a half years, producing the most alarming symptoms without warning, respiration suddenly ceased and the pulse became imperceptible. There was deadly palor. The child was inverted, and artificial respiration was put into practice. The time, he remarks, seemed to me to be hours, but which was nearly ten minutes, there was not the slightest response to our efforts. At length the application of cold water caused the child to make one little gasp; a second and third followed, and we soon desisted from our labors with a hearty sigh of relief. We have also found that in experimenting with small and young animals a heavy vapor of chloroform causes almost instant death. Another case of death from chloroform is reported in *British Medical Journal* in 1880, of a boy of seven, at University College Hospital, London. Another almost fatal case was reported to me by letter from Dr. F. C. Hotz, of Chicago, in a boy of eight years in an operation on the eye, and he concludes as follows: "I can assure you, Doctor, that I cannot describe the anxiety with which I watched the pupils, and how happy I was when I saw them contract again. I have still more cases of deaths both in young girls to report from chloroform. The first one occurred after a trifling operation on the eye by Prof. Jaeger, of Vienna. The bandage was applied, and the patient placed in bed when the heart ceased to beat, and breathing ceased with blueness of skin and death. A still more recent death occurred in Professor Bilroth's Clinic, Vienna, in an anemic girl in which the mixture employed was of chloroform and ether, and all efforts at resuscitation were fruitless, the girl died upon the operating table.

" We were once in doubt ourselves on this subject, but now we are fully convinced of the fact. We published eighteen such deaths, and since that time two more, and Dr. Kappeler has added two since, in all some twenty-two. Now, with regard to the use of chloroform in labor, we know of but few obstetricians who administer this agent to profound narcotism if they use it at all, and the few who employ it do not carry its effects further than the second stage of anæsthesea, and then finish the labor with ether." (Smith, Albert H., Philadelphia). Lusk says: "Deep anæsthesea carried to the point of complete abolition of consciousness, in some cases weakens uterine action, and sometimes suspends it altogether."

What is the opinion of Pajot, Pichand and Depaul, of France? They most decidedly oppose the use of chloroform, and Pichand who our reviewer calls " Richard," states in his fifth conclusion, " chloroform should never be pushed to complete insensibility" in labor. What is the opinion of surgeons like Sims, of New York, Elwood Wilson, of Philadelphia, or Byford, of Chicago? They will not use it in any operation in uterine surgery when they can obtain pure ether.

The subject was discussed last fall by the St. Louis Obstetrical Society, and considerable difference of opinion elicited. In the *Obstetrical Gazette*, Jan. 1881, Dr. D. M. Culver, of Indiana, believes that an anæsthetic often weakens the labor pains. He writes:

"I advise young physicians to use anæsthetics only in extreme cases —threatened convulsions or manual delivery—and let natural labors rest with nature's aid, chloroform. Doctor Reeves in his review states that we are prejudiced against chloroform, but such is not the fact, else we would never have published letters like those of Dr. L. B. Edwards, which Dr. R. quotes himself in favor of chloroform.

We have recorded a death from chloroform on the table by one of its warmest advocates, Dr. McGuire, of Richmond. Again we have to record another death from the South, as having occurred in Ballard County, Kentucky. The anæsthetic was administered for the removal of a wen upon the neck. The patient was thirty-five years old, six feet seven inches in height, and weighed two hundred and thirteen pounds. Cause of death, "heart disease." What has Bilroth to say in regard to the use of chloroform in his last German edition of his work? "Recently ether has come into use on account of the number of deaths from chloroform." Again, another death follows the administration of chloroform for medical purposes. It took place in the surgery of a dentist of Liverpool. The deceased being described by her regular medical attendant as of sound constitution. No blame, it is stated, can possibly be attached to any one, but the accident serves to point to the necessity for abandoning chloroform in favor of some anæsthetic not so often attended with fatalities. (3) *Medical Press and Circular*, Oct. 1880. In the recent discussion over the first death from ether in Cincinnati, there were reported by Drs. Dundride and Young cases of deaths from chloroform never before published by Dr. Musser.

We give a short abstract of the report on the action of anæsthetics to the scientific grants committee of the British Medical Association. The Committee's first report we have already referred to, the first in our work on anæsthetic. In conducting these investigations two lines were followed: first, to discover wherein the special danger of chloroform consists; and second, to attempt to find a safer anæsthetic. "Observations made on rabbits showed that chloroform had a most disastrous action on the heart, as well as upon the respiratory centre: that while ether might be employed for an indefinite period without affecting the heart, no sooner was the inhalation of chloroform commenced than the ventricle began to distend, and in course of time, the cardiac contraction ceased. In every respect but one ether was superior to chloroform. It had, however, one disadvantage—viz.: the length of time which was required to obtain its action.—*British Medical Journal*, Dec. 18, 1880.

### EROTIC EFFECT OF CHLOROFORM.

There is no anæsthetic that has been employed, that has produced the large number of cases, illustrative of this peculiar condition on the uterine organs, and we are firmly of the opinion that the number of such cases have been numerous, and, but few of them, unfortunately for the dental profession, have been published to the world from delicacy, but as sad, and fatal results have followed a want of knowledge by which a valuable life has been lost, and reputation and success in life have been blasted, such delicacy becomes false, and should be done away with, especially by the medical profession. Ether unless mixed with chloroform, rarely produces any erotic effect, and we have only been able to find one such case reported, which will be found in our work. Three authenticated cases from chloroform are given at p. 279, collected by us, and the fourth we will now give you:—

TRAGEDY IN A DENTAL OFFICE.—On July 26th, at about 5 o'clock, a terrible tragedy occurred in Oakland, California; the persons concerned being E. F. Schroeder, exchange teller in the London and San Francisco Bank, and Dr. Alfred Lefevre, a prominent dentist of Oakland. The latter was shot and killed by Schroeder, who visited him in his office, and fired two shots, one of which entered Dr. Lefevre's left side, ranging downward through the intestines and lodging in the opposite hip. The second shot missed, but the right side of the head and ear were powder-burned.

A San Francisco daily states that Mr. Schroeder has refused to make any statement regarding the affair, and the following particulars have been gathered from persons not directly interested:

According to the reports, it appears that Mrs. Schroeder, who is a daughter of Rev. Horatio Stebbins, of San Francisco, went down to the train on the day of the shooting, to meet her husband on his arrival from San Francisco. She was accompanied by her little daughter, some three years of age. Upon meeting her husband, Mrs. Schroeder is represented to have told him that on the Saturday previous, while under the influence of chloroform in Dr. Lefevre's office, a felonious assault had been made upon her by the dentist in question.

After hearing the story, Schroeder went with his wife and child to Dr. Lefevre's office corner of Eighth street and Broadway, and mounting the stairs, entered the Doctor's office. Advancing within a few feet of Lefevre, he pulled a pistol and fired two shots. One report states that Schroeder said, " Dr Lefevre, this is my wife ; take this," (firing) "and take that" (firing again.) The shooting took place at 4:50 o'clock, and Schroeder was immediately arrested. On the way to the City Prison, Schroeder said: " I hope to God I have killed him ; if I haven't I will. A man cannot seduce my wife and live."

Dr. Lefevre, upon being shot fell to the threshold of the door leading from the operating room to the private working room. He was raised and put upon a sofa and was soon surrounded by physicians who had been summoned. The wound was fatal, Dr. Lefevre survived about fourty minutes.

There are many people who believe that Mrs. Schroeder's charge against the deceased was purely illusory. It is well known that such hallucinations are not uncommon after the administering of chloroform. Some remarkable cases exist where hallucinations of this nature have taken the form of absolute conviction in the minds of the persons laboring under them, although there exist abundant evidence to prove that this conviction was utterly unfounded.

Dr. Lefevre was quite well known in Western New York, having been a student in the office of the late Dr. J. G. Barbor, of Le Roy, and went to California in 1862. We are informed that he was a native of Canada.

The coroner's jury rendered a verdict charging Schroeder with the murder of Dr Lefevre.

In the case of Schroeder for the murder of Dr. Lefevre, at Oakland, California, the jury, Dec. 19th, 1880, after being out seventy-two hours, returned a verdict of not guilty.

These peculiar delusions mentioned occur in the second stage of chloroform anæsthesia, and we have reported one case of our own personal knowledge. Dr. B. W. Richardson, of London, knew of a number of such cases, the truth of which results, were corroborated by the experience of Dr. Hawksby, of London, and by Dr. Saundby and Mr. I. F. West, of Birmingham, England; all medical men of high standing.

Our own observations on what is known as the mixed narcosis, morphia and chloroform, have been corroborated by the observations and experiments of "Demarquay," who claimed that the dangers of anæsthesia were by this method in no degree lessened—rather increased. The latest investigation of this method was by Heiter Konig, and the latter states he sees no difference between (the dangerous) chloroform and "morphia and chloroform" narcosis. In a table of 150 cases by Dr. Kappeler,* it appears the course of the anæsthetic with morphia is nearly the same as without it, yet he states: "I saw lately in the mixed narcosis, a patient, a woman thirty-six years old, with very dangerous symptoms on the part of the circulation, weak, irregular pulse, with pallor of the face, so that the administration had to be stopped;" and in his table of deaths from chloroform there is a case of fatal syncope in which "an injection of morphia preceded the inhalation."

### On The Safe Administration of Chloroform and Treatment of the Anæsthetic Narcosis of Chloroform.

---

The most recent suggestion as to the safe administration in prolonged operations is the now old idea of "Nassbaum," "Barnard," before referred to, and more lately by Dr. A. Crombia, in the PRACTITIONER, Dec., 1880, by which he reverses the usually recommended use of morphia by beginning the inhalation, and then immediately injects with the hypodermic syringe, twenty minims of the officinal solution of muriate of morphia, B. P. The advantage he claims for it is vomiting, more rare; chloroform asphyxia also seems to be prevented

*Anæsthetics, by Dr. O. Kappeler: Part XX. Surgery by Profs. Bilroth and Luecke, Stuttgart, 1880.

in two cases. Dr. C. has only noticed it once. Paralysis of the heart is less apt to happen, and he concludes it will be valuable in very warm climates, like India, etc., making chloroform as safe as ether, where ether cannot be employed. Dr. E. H. Jacob gives a word of warning in the January No. of the *British Medical Journal*, going over the same form we have done: he referred to the combined use of morphia and chloroform, which is open to less objection, than opium or morphia is when combined with the inhalation of ether, and yet neither free from danger, the one or the other agent, deepening the morphia narcosis or the morphia in preventing the patient from clearing his bronchi, from secretion provoked by the ether. "Several cases have recently come to Dr. Jacob's knowledge, in which death followed a short time after the administration of ether to a patient under the influence of opium. Dr. Albert W. Smith,† of Philadelphia, a warm advocate of the combined anæsthetics, had recently an almost fatal case from the use of ¼ grain muriate of morphia pellet and ether. Respiration fell to four or six per minute; pulse thready and slow; pupils almost invisible; she had indeed every symptom of extreme morphia poisoning, from which she only recovered with great difficulty. It required prolonged efforts at resuscitation, with artificial respiration, injections of coffee, and warmth and friction to the extremities."

### Chloroform Narcosis.

Schirmer, (Centralb-Augenf) claims to induce rapid recovery by irritating the nasal mucous membrane. It has long been known that the fifth nerve retains its sensibility longer than any other part in narcosis, and that inflexes may be induced through it when other irritations fail. Schirmer uses simply a roll of paper, which he turns in the nose. In bad cases he dips the paper in ammonia. We have found this method successful by applying the strong liquid ammonia to the nostrils. In this connection the nitrite of amyl must not be forgotten, for if promptly employed by the physician, by means of the nostrils it quickens the respiration, but its use, like many other agents, cardiac stimulants, must not be prolonged; like digitalis and atropine, which are useful in small doses, but if prolonged they

† "Philadelphia Times," March 26th, 1881.

retard the movements of the chest and lower animal temperature. In the hands of Dr. Hearn, the chief assistant of Professor Gross, nothing has yielded better results than the open chest smartly slapped with the fringe of a towel dipped in ice cold water. A piece of ice introduced into the rectum, and the head of the patient kept lowered, and the legs elevated. He also has employed ammonia to the nose at intervals, meanwhile an assistant practices artificial respiration and this must be kept up.*

Dr. F. W. P. Jago (*British Medical Journal*, December 11th, 1880) thinks that it would be proper to try *acupuncture of the heart*, by introducing a needle between the ribs near the apex of the heart, and pricking it slightly. He thinks it would be dangerous. (We feel sure it would be highly dangerous.)

Another plan he refers to is by *percussion of the heart*. He gives this instance:—

"Some time ago, a dentist, who had given bichloride of methylene, sent for me. I found a young and healthy-looking woman lying back, insensible, in the dentist's chair. The pulse and respiration had ceased for so alarming an interval that her case looked very bad indeed. Holding her wrist, to feel her pulse, it occured to me to give her one *sharp*, *very sudden* blow with my knuckles over the region of the apex of the heart. This appeared to produce the desired result: the patient gasped, drew a good inspiration, and a pulsation was at once felt at the wrist. But this is only one case; and, as one swallow is no proof of summer, it may not be a true instance of cause and effect after all; yet I feel sure that that sharp, rapid blow over the apex of the heart saved the patient's life."

Following this suggestion, another correspondent proposes stimulation by "Corrigan's button," a button shaped piece of metal heated in hot water and applied momentarily to the cardiac epigastric regions.

*See pages 104 and 303 Turnbull's Anæsthetic Manual.

(TO BE CONTINUED.)

